# Comparable area under the curve for three risk scores to detect interstitial lung disease in patients with rheumatoid arthritis: an external validation

**DOI:** 10.1007/s00296-025-06005-z

**Published:** 2025-10-15

**Authors:** Elin Blomberg, Bengt Wahlin, Anna Södergren

**Affiliations:** Department of Public Health and Clinical Medicine / Rheumatology, Umeå University, Umeå, 901 87 Sweden

**Keywords:** Rheumatoid arthritis, Interstitial lung disease, Risk assessment

## Abstract

Rheumatoid Arthritis associated Interstitial Lung Disease (RA-ILD) is an extraarticular manifestation of rheumatoid arthritis (RA) associated with increased morbidity and mortality. Several risk factors for RA-ILD have been identified, one is the promotor variant of mucin 5 B gene (MUC5B). Juge et al., Wheeler et al. and Koduri et al. have developed risk scores for identifying patients with RA at risk of RA-ILD. We aimed to externally validate the three risk scores and further investigate the frequency of MUC5B promotor variant and its association with subclinical lung changes in patients with RA in northern Sweden. Our cohort consisted of 54 patients with RA. The risk score variables were evaluated in binary logistic regression and validated using area under the receiver operating characteristics curve (AUC ROC). The genetic material was purified and genotyped for MUC5B. The Juge et al. risk score performed an AUC ROC of 0.71 (95% CI 0.57;0.86), the Wheeler et al. risk score an AUC ROC of 0.75 (95% CI 0.59;0.90) and Koduri et al. risk score an AUC ROC of 0.70 ((95% CI 0.55;0.85) in our cohort. The differences in AUC were not statistically significant. The MUC5B promotor variant frequency was 26% (*n* = 14). In our cohort, MUC5B was not significantly associated with subclinical lung changes. The three externally validated risk scores for RA-ILD performed well in this cohort and could be used clinically. In patients with RA in northern Sweden, MUC5B was not found to be independently associated with subclinical RA-ILD.

**Supplementary Information:**

The online version contains supplementary material available at 10.1007/s00296-025-06005-z.

## Introduction

Though joint involvement is central in rheumatoid arthritis (RA), it is a systemic illness with several extraarticular manifestations which contribute to increased morbidity and mortality in these patients [1, 2]. The majority of patients with RA have anti-citrullinated peptide-antibodies (ACPA). Smoking is associated with formation of ACPAs [3].

Cardiovascular and respiratory diseases are common causes of death among patients with RA [1, 4]. Interstitial lung disease (RA-ILD) constitutes a significant part of the morbidity and mortality in these patients [5]. It is known that high disease activity increases the risk for RA-ILD [6]. ILD can occur at any time during the RA disease course. Subclinical changes can be seen on high resolution computed tomography (HRCT) of the lungs before onset of respiratory symptoms [7].

It is described that declining diffusion capacity (DL_CO_) in pulmonary function tests (PFT) is highly sensitive for RA-ILD but lacks specificity due to high prevalence of emphysema in RA-ILD patients [8]. There is no common definition of RA- ILD; the diagnosis is usually based on HRCT, in combination with symptoms and decreased DL_CO_ on PFTs [6, 8–10].

Risk factors for RA-ILD are disease activity and high titres of ACPA, but also smoking, older age and male sex, which interestingly also are risk factors for idiopathic pulmonary fibrosis (IPF) [5, 6]. Potentially overlapping pathogenesis between RA-ILD and IPF is supported also by the shared common radiological pattern of usual interstitial pneumonia (UIP) [6, 8, 11]. Recent studies have found that a gain of function variant in the MUC5B promotor (rs35705950) is a both risk factor for UIP, the strongest genetical risk factor for IPF and a strong risk factor for RA-ILD [11–13].

Exacerbation of RA-ILD is a major cause of death in patients with RA-ILD [5]. The poor prognosis in RA-ILD highlight the need for an early diagnosis, and scores for estimating risk of ILD in patients with RA have been developed. for this purpose. Juge et al. developed a risk score for detecting subclinical RA-ILD including the variables sex, age at RA onset, RA disease activity measured by disease activity score based on 28 joints (DAS28) and MUC5B rs35705950 promotor variant [14]. This risk score has been externally validated in a large population of veterans with RA in a study by Wheeler et al. [15]. Wheeler et al. also developed and internally validated their own risk score including the risk factors age, sex, smoking status, DAS28- C-reactive protein (CRP), RF positivity and MUC5B rs35705950 promotor variant [15]. Koduri et al. [16] have, in a multicentre retrospective study, developed a clinical risk score including the variables age at RA onset, ever smoking, RF and ACPA. Definitions of RA-ILD varied between those studies, as did reasons for examining the patients included.

In the present study, we aimed to externally validate the three risk scores by Juge et al., Wheeler et al. and Koduri et al. to investigate whether they can predict subclinical lung changes in patients with RA in northern Sweden. We also aimed to determine the frequency of MUC5B promotor variant and analyse its association to lung changes in the same patients.

## Materials and methods

### Participants

All patients at the Department of Rheumatology in Umeå, Sweden, included in the Early Rheumatoid Arthritis Clinic (TRAM) were after 12 or 24 months of disease (during 2016–2017) invited to participate in a study of comorbidities in RA (*n* = 87) [17–20]. Inclusion and exclusion criteria have been previously described [17], but in short all patients ≤ 75 years of age and able to perform av submaximal exercise test were invited to participate. During 2019–2020, that is 4–7 years after diagnosis of RA, 55 of the 87 patients underwent HRCT of the lungs as well as PFT with measurement of diffusion capacity for carbon monoxide (DL_CO_). Thirty-two patients were excluded because of delay of the study due to the pandemic of COVID-19. Of the 55 patients that performed HRCT, one was excluded from further analyses due to incomplete inspiration when examined with HRCT. All participants fulfilled the 2010 American College of Rheumatology/ European League against Rheumatism Rheumatoid arthritis classification criteria [21]. The patients have been treated according to clinical practice. Evaluation of disease activity was done with DAS28 [22], Health assessment questionnaire (HAQ) [23], erythrocyte sedimentation rate (ESR) and CRP as a part of their clinical follow-up. Anti-CCP and RF were analysed at diagnosis using standard methods at the local department of immunology.

When risk scores were calculated, smoking was defined as current or former smoker. DAS28-ESR [22] and DAS28-CRP [24] were calculated. The participants had registered values of ESR, CRP, DAS28-SR, DAS28-CRP, and HAQ at diagnosis as well as after six, twelve and 24 months after diagnosis. Mean values of inflammatory variables over the first 24 months of disease included in the risk scores were calculated from these four assessments. Age at HRCT examination is the age reported. In Koduri et al. [16], age is divided into three age groups based on “age at RA onset”; < 40 years, 40–70 years and >70 years. Unlike Koduri et al. risk scores, the RF and ACPA variables in our material are dichotomous to seronegative and seropositive.

### Pulmonary examinations

HRCT of the lungs, PFTs and blood samples for genotyping and analysis of biomarkers were undertaken 2019–2020, i.e. approximately 5 years after RA diagnosis. HRCT was performed according to the Swedish CardioPulmonary BioImage Study (SCAPIS) protocol. The radiological images were assessed by an experienced radiologist and was interpreted according to the SCAPIS protocol [25]. In this study RA-ILD is defined as presence of honeycombing and/ or reticular pattern on HRCT.

PFT with dynamic spirometry and single breath determination of DL_CO_ was performed and analysed according to standard methods [26] and compared to Hedenström’s reference material [27]. Values below 95th percentile were classified as decreased.

### Genotyping

The genetic material was purified and genotyped at the local department of clinical genetics at Norrlands Universitetssjukhus in Umeå, using Reverse Transcriptase quantitative Polymerase Chain Reaction (RT qPCR) [28]. It was conducted according to TaqMan Genotyping Master Mix Protocol (TaqMan, Applied Biosystems, United States of America).

### Statistics

The data was analysed using SPSS version 29.0.1.0(171) (IBM SPSS Statistics Armonk, NY: IBM Corporation). Continuous variables are reported as mean and standard deviation (SD), and categorical variables are reported as frequency (n) and percentages (%). The variables included in the risk scores were analysed individually in univariable logistic regression models and together in multivariable logistic regression models. They were plotted in receiver operating characteristics (ROC) curves with a calculated area under the curve (AUC). Sensitivity, specificity and predictive values were calculated for the suggested cut off points according to the publications on the risk scores. To statistically compare AUC for the risk scores, paired sample area difference under the ROC curves was used. An AUC between 0.7 and 0.8 was considered fairly well, AUC between 0.8 and 0.9 good and over 0.9 excellent [29]. R^2^ in logistic regression refers to the Nagelkerke R^2^ value. Pearson’s chi-squared test (Chi2) was used when appropriate. P-values below 0.05 was considered statistically significant.

### Missing values

Missing values are regarded as random based on analysis of patterns of missing values. To be able to calculate risk scores despite missing values, multiple imputation was performed based on the variables ACPA titre, ESR, CRP, Visual analogue scale (VAS) global assessment, VAS pain, VAS fatigue, and number of tender and swollen joints creating 10 datasets. The mean values of the imputed values were used in further analyses [30].

### Ethics

The study was 2018-02-22 approved by the regional board of ethics at Umeå university (2017-500-31 M).

### Consent to participate

Informed consent was obtained from all individual participants included in the study.

## Results

### The participants

Fifty-four patients with RA were included (Table 1). All participants had in some extent missing data. Of the values used in this study, there was initially 7.5% missing data. After performing multiple imputation, missing data was reduced to 1.1%. The frequency of subclinical ILD in our material was 26% (*n* = 14) (Table 1).

### Frequency of MUC5B promotor variant rs35705950

The frequency of MUC5B promotor variant was 26% (*n* = 14) (Table 1).

### Association between MUC5B promotor variant and lung changes

A Chi2 test with MUC5B T-allele and RA-ILD defined as reticular opacities and/or honeycombing showed a prevalence of MUC5B of 43% in individuals with RA-ILD, whereas the prevalence was 20% in individuals without ILD, but the difference was not statistically significant (p 0.09) (Table 1). No statistically significant association was seen in univariable binary logistic regression with MUC5B as covariable and RA-ILD as dependent variable (OR = 3.0; 95% CI 0.81–11.1; R^2^ = 0.07).

When widening the definition to lung changes defined as reticular opacities and/or honeycombing and/or ground glass opacities, there was still no statistically significant differences in prevalence of MUC5B T-allele ( OR = 2.33; 95% CI 0.67–8.1; R^2^ = 0.04).

### Risk score validation

**Fig. 1:**
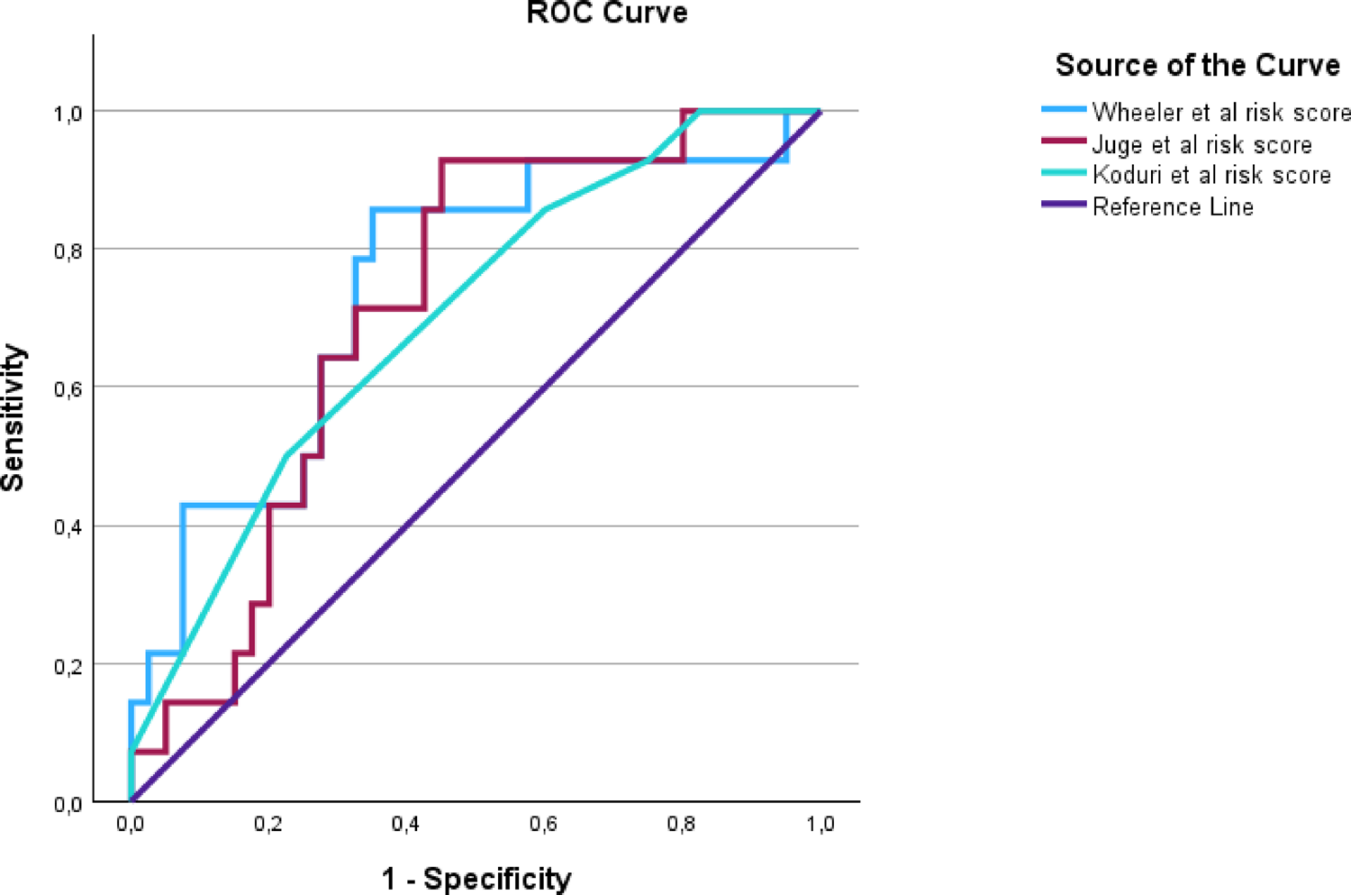
In patients with RA in northern Sweden, the risk scores from Juge et al. [14], Wheeler et al. [15] and Koduri et al. [16] were valididated using ROC curves for the binary outcome of RA-ILD defined by honeycombing and/ or reticular pattern on HRCT. All three ROC curves are presented with sensitivity on the y-axis and 1- specificity (false positive rate) on the x-axis. The Wheeler et al. risk score ROC curve is closest to the upper left corner. *RA* rheumatoid arthritis, *ROC* receiver operator characteristics, *HRCT* high resolution computed tomography *AUC* area under the curve. The figure is created using SPSS version 29.0.1.0

The risk scores were calculated and validated as described in Methods. Risk score calculations are presented in Supplement. The ROC curves of the respective risk scores are presented in Fig. 1.

### Juge et al. risk score

AUC for Juge et al. risk score was 0.71 (95% CI 0.57–0.86). The variables were evaluated in logistic regressions and age was statistically significantly associated with RA-ILD (Table 2).

The sensitivity and specificity as well as positive and negative predictive values were calculated for the suggested cutoff of 51. Of the included patients, 92.6% (*n* = 50) were above the cutoff for RA-ILD and 7.4% (*n* = 4) were below the cutoff. The sensitivity in our material was 100% and the specificity was 10%. Positive predictive value was 28% and negative predictive value 100%.

### Wheeler et al. risk score

AUC for Wheeler et al. risk score was 0.75 (95% CI 0.59–0.90). The variables were evaluated in logistic regressions and age was statistically significantly associated with RA-ILD (Table 2).

For the suggested cut off 0.05, the sensitivity in our material was 93% and specificity was 40%. Of our patients, 68.5% (*n* = 37) were above the cutoff and 31.5% (*n* = 17) were below the cutoff. The positive predictive value was 35% and negative predictive value 94%.

### Koduri et al. risk score

AUC for Koduri et al. risk score was in our material 0.70 (95% CI 0.55–0.85). The variables were evaluated in logistic regressions and age as a continuous variable was statistically significantly associated with RA-ILD (Table 2).

Koduri et al. set the cut off value to 5 to divide into high and low risk of RA-ILD. The sensitivity was in our material 50% and the specificity was 77.5%. Of our patients, 29.6% (*n* = 16) were above the suggested cut off, whereas 70.4% (*n* = 38) were below the cut off. The positive predictive value was 43.8% and negative predictive value 82.6%.

To compare the three risk scores, paired sample area difference under the ROC curves were used. No statistically significant difference was found (data not shown).

## Discussion

In this study three external risk scores [14–16] for predicting risk for RA-ILD were validated in patients with RA in northern Sweden. All three risk scores performed fairly well in our material without statistically significant differences in discrimination, although sensitivity, specificity and predictive values differed. Risk scores could be a valuable tool in early diagnosis of RA-ILD in order to reduce morbidity and mortality in these patients. Finding high risk patients early and offering them evaluation and strategies to prevent development of RA-ILD would be groundbreaking, and it would also be beneficial to rule out low risk patients from extensive follow up, to reduce costs and exposure for ionized radiation. A recent Turkish study of screening methods for RA-ILD did not include risk scores, indicating that they are uncommon in clinical practice [29].

The Juge et al. risk score [14] performed a lower AUC ROC (0.71) for detecting subclinical RA-ILD in our material compared to AUC ROC of 0.82 in the developing population. In Wheeler et al., where RA-ILD was defined as a clinical diagnosis, the Juge et al. risk score was validated with an AUC of 0.658 [15]. The Juge et al. risk score thus performs better in our population than in the population analysed by Wheeler, where RA-ILD outcome was not subclinical. An AUC of 0.71 can be interpreted as a fairly well model for prediction [30]. The Juge et al. risk score was developed in patients with a mean RA duration of 10 years, whereas in our material the participants had a disease duration of approximately five years. This difference in disease duration is unlikely to perturb the results. It has been described that 10% of RA patients have ILD after two years of disease duration [6], whereas in a study by Hyldgaard et al., 14% of RA-ILD patients was diagnosed 1–5 years before their diagnosis of RA [4]. These numbers suggest that RA-ILD is present very early in the course of disease, making it plausible that differences in disease duration between the populations studied do not have a large impact on results when comparing the performance of risk scores in different populations.

The sensitivity of the Juge et al. risk score in our population was 100% compared to 75% in the developing population. The specificity was on the other hand estimated to only 10% in our population, compared to 85% in their population. Since the risk score was developed to detect subclinical RA-ILD and the patients in the present study were asymptomatic, this difference is surprising. This risk score is in our population inadequate in ruling out people without subclinical RA-ILD - the risk score only ruled out 7.4% (*n* = 4) of our participants as low risk of RA-ILD.

The Wheeler et al. risk score [15] performed a higher AUC ROC in our material compared to AUC ROC of 0.68 in the large discovery population of veterans with RA (*n* = 2386). The sensitivity was similar in our material (93% compared to 92% in the development population) and the specificity was higher (40% compared to 22%) for the suggested cut off 0.05, although the participants in the development population were symptomatic. The participants in the development population were mainly men (89.2%) while the present study, as usual when individuals with RA are studied, had an overweight of women (64%). The prevalence of subclinical RA-ILD was in the present study higher in men than women. This is congruent with current knowledge that men have a higher risk of RA-ILD than women [5, 8–10].

The Koduri et al. risk score differs from the other two by being based on easy accessed clinical variables. That risk score performed a slightly lower AUC ROC in our material (0.70) compared to the AUC ROC of 0.76 in the discovery population [16]. The variables RF and ACPA were not used entirely according to Koduri et al., as previously mentioned. The already basic risk score thus became even more rough, which may not be to its advantage in discriminating between individuals with low and high risk of RA-ILD. This risk score performs the lowest AUC of the risk scores included in the present study, yet the difference is not statistically significant. The shape of the curve is relatively flat and is the furthest from the upper left corner of the ROC curve (Fig. 1). This is mirrored in the low sensitivity of 50% at the suggested cut off at 5. This risk score however performs a higher specificity in our material than in the discovery population [16].

When comparing the performance of the three risk scores, the Wheeler et al. risk score performs the highest AUC ROC, and the curve is closest to the upper left corner of the ROC diagram. The sensitivity is high for both the Juge and Wheeler et al. risk scores, but the Wheeler risk score has higher specificity. The Koduri et al. risk score performs the lowest AUC, has the lowest sensitivity, but has the highest specificity. The Koduri et al. risk score could be considered the best at ruling out people at low risk of RA-ILD but inadequate in finding RA-ILD. The Wheeler risk score, on the other hand, has been developed in the largest population and could be assumed to have better transferability and generalizability and less issues with overfitting. The difference between them is however not statistically significant in our population.

In our material the number of individuals with RA-ILD and the risk allele (GT/TT) was low. Given the small material, it is difficult to determine whether this can be generalised for the whole RA population in northern Sweden. The allele frequency in the Juge et al. study was 11.3% [14]. In a multiethnic case series, the allele frequencies vary among RA patients in different countries [11] - the allele frequency in France was 23.3% and in Mexico 9.3%. In two separate case series from the United States the allele frequency was 21.1% and 12.9% respectively, but only 0.7% in Japan [11]. Wheeler et al. had an allele frequency of 20.1% among veterans with RA in the USA [15]. The low allele frequency in Japanese patients align with other studies demonstrating that the MUC5B promotor variant is low in Asian populations. In Joo et al. the allele frequency in Korean patients with RA was 0.37%, yet still significantly associated to RA-ILD with UIP pattern [32]. In a Chinese study by Wang et al., the allele frequency in individuals with IPF as 2.22% and statistically significantly associated to IPF but not to connective tissue disease associated-ILD [33]. This implies that MUC5B promotor variant is associated to fibrosing lung disease despite large differences in allele frequencies across different ethnical populations. In low frequency populations the clinical relevance of analysing MUC5B may be limited, but it is important in regards of elucidating the pathogenesis of ILD. Our study consists of representative individuals with RA in northern Sweden and despite the small sample size, the allele frequency is probable, based on the allele frequency of other populations discussed above. Still, in contrast to other studies [5, 11–15, 34], the MUC5B promotor variant was not statistically significant associated with ILD in our study, although the frequency was higher in patients with RA-ILD.

This study has some limitations. The first is the small study population. The power calculation was based on the aims of studying comorbidities [17–20], and further reduced by the COVID-pandemic, making the number of included participants small. Another limitation is the absence of a general RA-ILD definition, both in the development of the included risk scores and in the whole research area. In this study RA-ILD is defined by HRCT findings of pulmonary fibrosis, as specified in a predefined protocol [25]. Moreover, this study validated risk scores developed in other populations and included only risk scores where data on all variables for the risk score was available in our material. The study was also single-center, reducing the generalizability of the results, and possible confounders like pharmacological treatment, comorbidities and other risk factors for ILD were not included in the analyses.

One of the major strengths of this study is that the material is well characterized, since the participants were included in a larger study aiming to study physical activity and comorbidities in patients with RA [17–20]. The material is unselected since all patients with RA diagnosis, age over 18 years and being able to perform the examinations were invited. This reduces the risk of selection bias. Another strength is that multiple imputation was performed to maximise the use of available information. Multiple estimations of probable values for missing values based on preexisting values is superior to single imputations methods. However, a disadvantage with multiple imputation is the randomness in the estimates of the probable value which makes it hard to reproduce the exact same imputed values if performed again [30]. However, this has been considered and the number of estimates was increased to 10 from the default of 5 to increase the certainty of the estimates.

Three risk scores for RA-ILD have been externally validated with fairly good results regarding ROC curves and AUC in our population. The allele frequency of MUC5B was similar to the reported allele frequencies in other European populations, but unlike many other studies MUC5B was not statistically significant associated with lung disease, although there was a numerical association. Taken together, further studies are required on developing risk scores for RA-ILD to increase specificity without compromising sensitivity, maybe using biomarkers along with the variables included in the present risk scores.

**Table 1:** Demographic data of 54 patients with RA from Northern Sweden

Characteristics	All patients *n* = 54	RA-ILD *N* = 14	No RA-ILD *N* = 40
Demographics			
Women, n	34 (63%)	7 (50%)	27 (68%)
Age, years	60.9 (13.7)	69.1 (7.3)	58.1(14.4)**
BMI, kg/m^2^	27.1 (4,6)	27.5 (5,2)	26.9 (4,5)
Ever smoker, n	25 (46%)	9 (64%)	16 (40%)
RA specifics			
aCCP positivity, n	36 (67%)	11 (79%)	25 (63%)
aCCP titer, units/ml #	149.3 (175.7)	238.7 (231.3)	118.0 (142.3)*
RF positivity, n	39 (72%)	12 (86%)	27 (68%)
ESR mean first 24 months, mm/h #	19.0 (10.8)	25.4 (10.5)	16.8 (10.1)**
CRP mean first 24 months, mg/l #	8.0 (7.7)	10.9 (7.6)	6.9 (7.6)
DAS28-ESR mean first 24 months #	3.4 (0.9)	3.4 (0.5)	3.3 (1.0)
DAS28-CRP mean first 24 months #	3.1(0.8)	3.0 (0.5)	3.1 (0.9)
HAQ mean first 24 months	0.60 (0.40) *n* = 46	0,68 (0.45) *n* = 12	0,56 (0.39) *n* = 34
MUC5B rs35705950, n	14 (26%)	6 (43%)	8 (20%)
HRCT findings			
Reticular patterns, n	14 (26%)	14 (100%)	0 (0%)***
Honeycombing, n	2 (4%)	2 (14%)	0 (0%)*
Ground glass opacities, n	18 (33%)	13 (93%)	5 (13%)***
Pulmonary functions tests			
FVC, L	3.75 (1.00)	3.23 (0.88)	3.94 (0.98)*

RA-ILD is defined as honeycombing and/or reticular pattern in HRCT. Continous variables are reported as mean (standard deviation), whereas categorical variables are reported as frequency (percentage). Variables including imputed data are marked with #

*RA* rheumatoid arthritis, *RA-ILD* rheumatoid arthritis interstitial lung disease, *HRCT* high resolution computed tomography, *BMI* body mass index, *aCCP* anti cyclic-citrullinated peptide antibodies, *RF* rheumatoid factor, *ESR* erythrocyte sedimentation rate, *CRP* C- reactive protein, *DAS28* disease activity score 28 joints, *HAQ* health assessment questionnaire, *MUC5B* mucin 5 B, *FEV1* forced expiratory volume 1 s, *FVC* forced vital capacity

**Table 2:** Logistic regression analysis in 54 patients with RA in Northern Sweden with RA-ILD as dependent variable

Variable	Univariable logistic regression OR (CI)	*R* ^2^ univariable regression	Model 1 = Juge et al. [14] OR (CI)	Model 2 = Wheeler et al. [15] OR (CI)	Model 3 = Koduri et al. [16] OR (CI)
Age at examination, year	**1.09 (1.01;1.17)**	0.21		1**.09 (1.01; 1.19)**	
Age at RA onset, year	**1.078 (1.01;1.15)**	0.18	**1.08 (1.01;1.16)**		**1.09 (1.01; 1.17)**
Male sex, yes	0.48 (0.14;1.66)	0.036	1.65 (0.38; 7.24)	1.51 (0.30; 7.50)	
Ever smoker, yes	2.70 (0.76;9.55)	0.066		1.02 (0.21; 4.95)	1.41 (0.34; 5.90)
MUC5B risk allele, yes	3.0 (0.81; 11.14)	0.070	3.37 (0.76; 14.8)	4.18 (0.82; 21.8)	
DAS28-SR mean value first 24 months	1.11 (0.57; 2.16)	0.003	1.11 (0.45; 2.72)		
DAS28-CRP mean value first 24 months	0.85 (0.38; 1.86)	0.005		0.85 (0.31; 2.33)	
Poisitve RF, yes	2.89 (0.56; 14.8)	0.050		0.85 (0.53; 21.8)	2.1 (0.33; 13.8)
Positive aCCP, yes	2.2 (0.53; 9.2)	0.034			2.39 (0.46; 126)
FVC, liter	0.44 (0.21; 0.92)	0.15			
ESR mean first 24 months, mm/H	1.08 (1.02;1.15)	0.17			
R^2^ multivariable regression			0.26	0.33	0.26

The independent variables included in the risk score models are included in the univariable and multivariable logistic regression models. The data is presented as odds ratio (OR) and 95% confidence intervals (CI). The R^2^ value is Nagelkerke R^2^ value

*RA* rheumatoid arthritis, *RA-ILD* rheumatoid arthritis interstitial lung disease, *aCCP* anti cyclic-citrullinated peptide antibodies, *RF* rheumatoid factor, *ESR* erythrocyte sedimentation rate, *CRP* c- reaktive protein, *DAS28* disease activity score 28 joints, *MUC5B* mucin 5 B, *FVC* forced vital capacity

## Supplementary Information

Below is the link to the electronic supplementary material.

Supplementary Material 1

## Data Availability

Data will be shared upon reasonable request.
