# Supplementary material for: Comparable area under the curve for three risk scores to detect interstitial lung disease in patients with rheumatoid arthritis: an external validation
Source: Rheumatol Int. 2025 Oct 15;45(11):252. doi: 10.1007/s00296-025-06005-z (PMC12528251; doi:10.1007/s00296-025-06005-z)
Supplement: Supplementary file 1 — Supplementary Material 1 [file 296_2025_6005_MOESM1_ESM.docx]

**Supplement**

*Formulas of the external risk scores*

To validate the risk score provided by Juge *et al.* [14] the following formula was used. The coefficients multiplied with the variables are the odds ratio presented by Juge for each variable respectively:

Juge *et al.* risk score= ((3.74 * MUC5B rs35705950) +(3.93 * male sex) +(1.1 * age at RA onset) + (2.03* mean DAS28))

The formula for the risk score produced by Wheeler *et al.* [15] was calculated according to the instructions in their supplementary material.

First step: Calculated the model constant/logit: ((ever smoker * 0.3786118) +((age-69.5) * 0.0253954) +(male sex * 0.301797) +(mean DAS28-CRP * 0.2384218) +(RF seropositivity *0.63059) +(MUC5B rs35705950 *0.8498984))-4.389373

Second step: Probability of RA-ILD, calculated: e^logit^/(1+e^logit^).

The formula for Koduri *et al.* four-factor risk score is based on categorical variables in multivariable regression where the odds ratio constant has been replaced by a given weighting coefficient (0,1 or 2) [16].

Probability for ILD: ((age at RA onset (groups)) * weighting coefficient)) + (ever smoker * weighting coefficient) + (ACPA positivity * weighting coefficient) + (RF positivity * weighting coefficient)
